# 8 A Multimodal Critical Care Pediatrics Course Improves Nursing Knowledge and Comfort

**DOI:** 10.1093/jbcr/iraf019.008

**Published:** 2025-04-01

**Authors:** Courtney Lawrence, Emily Daniels, Kristy Gauthier, Christopher LaChapelle, Giavonni Lewis, Crystal Webb, Callie Thompson

**Affiliations:** University of Utah Health Burn Center; University of Utah Hospital; University of Utah, College of Nursing and Burn Center; University of Utah Health Burn Center; University of Utah Health Burn Center; University of Utah Health Burn Center; University of Utah Health Burn Center

## Abstract

**Introduction:**

Intubated pediatric patients with thermal injuries are a low-frequency patient population that are at a high risk for complications. Our burn center noted an unacceptable rate of medication errors, emergent procedure complications, and issues regarding ventilator management. Upon investigation, bedside nurses reported they feel underprepared to manage these patients with the existing orientation structure: hands-on training, paired with an expert nurse, for six twelve-hour shifts.

**Methods:**

A didactic course & simulation-based assessment was developed through current evidence-based practice & a collaborative effort with many departments and disciplines, including attending physicians; nursing management; nursing education; clinical nurse experts; respiratory therapy & pharmacy. The course was then trialed with expert RNs with pre- and post-testing which demonstrate efficacy and utility.

The course was then expanded and implemented to non-expert RNs who were preparing to take care of intubated pediatric patients. Didactic material was expanded and presented in-person by an interdisciplinary team. Simulation is held in an advanced simulation lab with a high-fidelity pediatric mannequin. The simulation was expanded to include participants from an interdisciplinary team including RNs, respiratory and Burn APCs.

Learners completed pre- and post-surveys including a self-assessment of the education they have been provided as well as their knowledge to safety and competently take care of a critical pediatric patient.

**Results:**

We have held four courses: two in 2023 (13 RNs) and two in 2024 (17 RNs). We sent a pre-course survey to 17 RNs in 2024, received a 100% response. 2 RNs did not complete the course, & the remaining 15 RNs were sent a post-survey, received a 100% response.

A comparison of pre and post surveys showed a large increase in the % of respondents feeling they have been provided the appropriate education to safely care for pediatric patients, 12% to 76%. Respondents also indicated an increase in adequate pediatric knowledge with an increase from 12% to 70%.

**Conclusions:**

The didactic material & simulation of this critical care peds course has been a valuable addition to our nursing staff prior to their hands-on orientation with an expert nurse with an intubated pediatric burn patient. This course has allowed for staff to be better prepared for high-intensity, real life, situations involving the intubated pediatric population.

**Applicability of Research to Practice:**

High nursing turnover and relatively inexperienced nursing staff has been a challenge for burn centers for many years. Here we describe a new educational effort used to improve new nurses’ comfort with a high-stress patient population. This course can be scaled and implemented to other burn centers.

**Funding for the Study:**

N/A

